# Introducing OpenTextile-NIR: Near-infrared hyperspectral imaging and photography dataset for optical identification of textiles

**DOI:** 10.1016/j.dib.2026.112559

**Published:** 2026-02-09

**Authors:** Tuomas Sormunen, Ella Mahlamäki, Satu-Marja Mäkelä, Mikko Mäkelä

**Affiliations:** a VTT Technical Research Centre of Finland Ltd., Kaitoväylä 1, Oulu 90570, Finland; b VTT Technical Research Centre of Finland Ltd., Tietotie 4E, Espoo 02150, Finland

**Keywords:** Near infrared spectroscopy, Garments, Textile waste, Recycling, Sorting, Machine learning

## Abstract

This dataset presents the first open-access collection of near-infrared hyperspectral imaging (NIR-HSI) data for the optical identification of textiles, with a focus on supporting research in sensor-based textile sorting and recycling. The dataset comprises hyperspectral images, RGB photographs, and detailed metadata, including fibre composition and colour, for 71 post-industrial textile samples, collected in Finland. Over 11 million spectra are included in the hyperspectral images, with more than 6 million annotated, providing a robust foundation for machine learning and data analysis. In addition, we provide a single representative NIR spectra and RGB value for each sample in order to accommodate classic spectroscopic analysis.

Used garments were sourced from a partner company specializing in end-of-life textile management, with ground truth information on fibre composition obtained from suppliers. Small pieces of each garment were measured using Specim SWIR 3 hyperspectral camera and photographed with high-resolution mobile phone camera (Samsung Galaxy A52). The dataset is organized into folders containing raw and processed data, including ENVI-format hyperspectral images, RGB images, as well as CSV files with mean spectra, mean RGB values, and sample metadata. An example Python script is provided to facilitate data access and processing.

Potential reuse scenarios include classification of textiles by material or colour, prediction of natural fibre content, image segmentation, algorithm development for spectral classification, and use as a reference spectral library. The dataset’s comprehensive structure and open availability address the limitations of previous research, which often relied on small or non-public datasets, and is intended to accelerate advances in optical identification technologies for textile recycling.

Specifications Table

**Table:** 

Subject	Engineering & Materials science
Specific subject area	Optical identification of different garments for sorting based on various features textile features such as fibre composition and colour.
Type of data	Image, Chart, ENVI Raw, Processed
Data collection	Used garments were collected by a partner company specializing in end-of-life management of post-industrial textiles. Ground truth information of the samples was gathered by the partner company from their suppliers. Small pieces were cut from these garments and were measured using near-infrared hyperspectral imaging (Specim SWIR 3) and photography (Samsung Galaxy A52).
Data source location	All the samples and data were collected in Finland. The data is stored at the authors’ institution.
Data accessibility	Repository name: Zenodo Data identification number: [DOI: 10.5281/zenodo.18269172] Direct URL to data: [doi.org/10.5281/zenodo.18269172] Instructions for accessing these data: Zenodo is free and open access to use without the need for registering or other special requirements.
Related research article	none

## 1 Value of the Data

• Textile recycling is currently heavily under research and development to deal with the huge amounts of generated textile waste. Different recycling techniques have different requirements in terms of what can be input to each process. Since the variability of fibres and fibre blends in textiles is wide, accurate sorting according to fibre type is required in order to generate high-quality feedstock. Currently, textile sorting is done manually, but in order to scale-up the process, automated technologies are needed. Arguably, one of the most promising technology in this domain is optical sorting, which is expected to break through in the near future. However, this technology requires very high number of reference sample data to be able to answer to the sorting requirements.
• Our dataset presented in this paper fills a critical gap by providing the first open-access near-infrared hyperspectral imaging (NIR-HSI) dataset of textiles, supporting research and development in optical textile identification, sorting and recycling. NIR-HSI is seen as a key enabler in textile sorting due to the fact that it can, based on the spectral response, identify different fibre and fibre blends in a piece of textile. Since NIR-HSI data contains spatial dimension in addition to the spectral dimension, different garment components made of different material can be identified as well.
• Our dataset is comprehensive, as it contains hyperspectral images, photographs, and detailed metadata (including fibre composition and colour) for 71 post-industrial textile samples, with over 11 million spectra, more than 6 million of which are annotated, enabling robust material for machine learning and data analysis. The dataset can be reused for tasks such as classification of textiles by material or colour, prediction of natural fibre content, image segmentation, algorithm development for spectral classification, and as a reference spectral library for different textile materials. Unlike most prior research, which used small or non-public datasets, this resource offers a large, well-documented, and openly shared dataset, increasing its relevance and impact for the scientific community and industry alike.
• Researchers, optical sensor and sensor-based sorter manufacturers, algorithm developers, waste management companies and textile recyclers may benefit from our dataset. For researchers, the dataset provides material for spectroscopic analysis. For sensor and sensor-based sorter manufacturers, the dataset provides reference spectra which can be used in finding optimal wavelength ranges for identification and thus selection of appropriate sensor technology. For algorithm developers, the contained data can be used for testing different pre-processing and machine learning methods. Finally, for waste management and textile recylers, the dataset provides information on the capabilities of the used sensor technologies in automated sorting.

## 2 Background

Recycling is necessary to deal with ever-increasing amounts of textile waste, but it requires accurate sorting according to fibre type, which is often done manually. There has been growing interest in optical identification of textiles, enabling sensor-based sorting. Most published research articles in this domain deal with very limited sample sets, either in terms of quantity or variability. Moreover, the datasets are rarely public, limiting their relevance.

Some open datasets related to textiles are available. NIST recently published near-infrared (NIR) spectra and optical microscopy data of 64 virgin and post-consumer fabrics [1]. RISE published photographs of over 31000 post-consumer garments from sorting facility [2]. UEF published visible hyperspectral imaging textile texture database [3]. TextileNet contains web-crawled images of clothes and associated labels [4], and finally, the dataset by Gil-Arroyo [5] is related to textile defect detection. To the best of our knowledge, these datasets are the only ones available.

Near-infrared hyperspectral imaging (NIR-HSI) has recently gained popularity as an imaging modality. This method is seen as a key enabler for sensor-based sorting, as it allows for pixel-by-pixel NIR analysis over a large area. As of yet, no NIR-HSI datasets of textiles are openly available. This article bridges this gap.

## 3 Data Description

The dataset contains hyperspectral images and photographs of 71 post-industrial textile samples as well as metadata of each sample, including the fibre composition and colour. HSI in essence combines spectroscopy with spatial data. In spectroscopy, a sensor is used to measure the absorbance of electromagnetic radiation by a sample on different wavelengths, generating a spectrum. This absorbance is dependent on the molecular composition of the sample. Organic molecules are very active in the near-infrared range, with near-infrared spectroscopy enabling identification of different organic components in a sample. As opposed to spectroscopy in which a single point is measured, HSI measures a large area, with each pixel comprising of a spectrum.

**Fig. 1:**
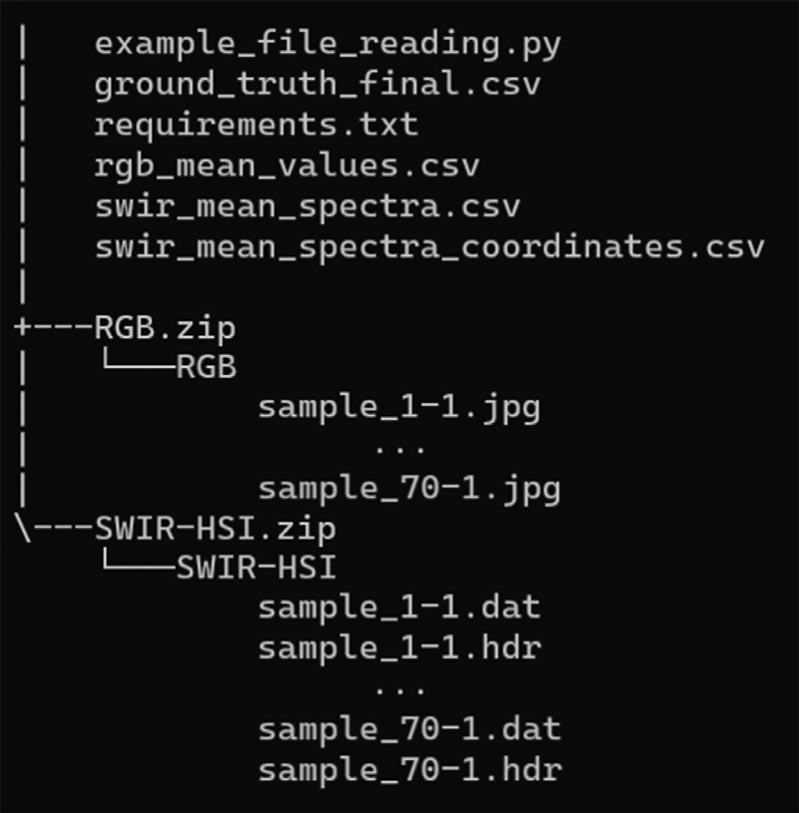
Visualization of the dataset structure.

The size of the dataset is approximately 11.8 gigabytes. It contains over 11 million spectra, over 6 million of which are annotated. The red-green-blue (RGB) photographs contain over 243 million pixels, of which over 26 million are annotated. The samples are labelled with “sample_NNNN”, for example, “sample_15-1”. The dataset is structured as seen in Fig. 1. In this section, we give a detailed description of each file and folder.

### 3.1 File: example_file_reading.py

The details of this file are as follows:

• Python script file
• Contains an example script of how to read into memory each of the different files in this repository, as well as how to extract the region of interest (ROI) of RGB and NIR-HSI images of an example sample.
• Developed with Python 3.10.11^1^, on 64-bit Windows 10

### 3.2 File: ground_truth_final.csv

The details of this file are as follows:

• Comma separated value (csv) file, semicolon separator “;”
• Contains information of each sample, including composition and appearance
• Contains 72 rows (including one header row)
• Contains 14 columns (including one index column):
  ○ Label: label of the sample, in format “sample_NNNN”
  ○ Unknown or outlier?: determines whether the composition of the sample is unknown (row value equals “unknown”), or whether it is an outlier based on our data analysis (row value equals “outlier”). If empty, the composition is known and the sample is not deemed an outlier
  ○ Composition columns, 11 in total:
    ■ Polyamide [%]: percentage of polyamide in the sample
    ■ Viscose [%]: percentage of viscose in the sample
    ■ Lyocell [%]: percentage of lyocell in the sample
    ■ Modacrylic [%]: percentage of modacrylic in the sample
    ■ Polyacrylic [%]: percentage of polyacrylic in the sample
    ■ Wool [%]: percentage of wool in the sample
    ■ Carbon fibre [%]: percentage of carbon fibre in the sample
    ■ Polyurethane [%]: percentage of polyurethane in the sample
    ■ Elastane [%]: percentage of elastane in the sample
    ■ Polyester [%]: percentage of polyester in the sample
    ■ Cotton [%]: percentage of cotton in the sample
  ○ Appearance columns, 2 in total:
    ■ Color: main colour of the sample
    ■ Texture: If the sample contains textural features such as stripes or grids, this information is given in this column. Otherwise, this column is empty
  ○ Extra information: Extra information of the sample that does not concern other columns

### 3.3 File: requirements.txt

The details of this file are as follows:

• Text file containing the library packages required for running the “example_file_reading.py” Python script
• The packages can be installed with e.g. pip package installer using the following command (without quotation marks): “pip install -r requirements.txt”
• Used PIP version: 23.0.1^2^

### 3.4 File: rgb_mean_values.csv

The details of this file are as follows:

• Comma separated value (csv) file, semicolon separator “;”
• Contains the mean RGB values of each sample
  ○ The region of interest from which the mean value is calculated is the same for all samples, i.e. a rectangle of size 615 pixels by 602 pixels encompassed by the following pixel coordinates:
    ■ y_start, y_end = 564, 1180
    ■ x_start, x_end = 675, 1278
• IMPORTANT: the pixel coordinates conform to the Python syntax, where the starting index is 0, and the end column and row (y_end, x_end) are NOT included in the region of interest
• Contains 72 rows (including one header row)
• Contains 4 columns (including one index column):
  ○ Label: label of the sample, in format “sample_NNNN”
  ○ R: mean R color value of the sample
  ○ G: mean G color value of the sample
  ○ B: mean B color value of the sample

### 3.5 File: swir_mean_spectra.csv

The details of this file are as follows:

• Comma separated value (csv) file, semicolon separator “;”
• Contains the mean NIR spectrum of each sample.
  ○ The region of interest from which the mean value is calculated is unique for each sample, and are enumerated in the file “swir_mean_spectra_coordinates.csv”
• Contains 72 rows (including one header row)
• Contains 289 columns (including one index column):
  ○ Label: label of the sample, in format “sample_NNNN”
  ○ The remaining 288 columns contain the reflectance values of each wavelength, in the range 953.04 – 2547.64 nanometres; the wavelengths are enumerated in the header row

### 3.6 File: swir_mean_spectra_coordinates.csv

The details of this file are as follows:

• Comma separated value (csv) file, semicolon separator “;”
• Contains the region of interest from which the mean NIR spectrum of each sample is calculated
○ The mean values themselves are contained in the file “swir_mean_spectra.csv”
• Contains 72 rows (including one header row)
• Contains 5 columns (including one index column):
  ○ Label: label of the sample, in format “sample_NNNN”
  ○ y_start: starting y-coordinate of the region of interest
  ○ y_end: ending y-coordinate of the region of interest
  ○ x_start: starting x-coordinate of the region of interest
  ○ x_end: ending x-coordinate of the region of interest
• IMPORTANT: the pixel coordinates conform to the Python syntax, where the starting index is 0, and the end column and row (y_end, x_end) are NOT included in the region of interest

### 3.7 Folder: RGB.zip

This .zip archive^3^, named “RGB.zip”, contains a folder named “RGB”. The details of this folder are as follows:

• Folder containing photographs of the samples
• File names in format “sample_NNNN”
• Image format: jpg
• Constant image dimensions:
  ○ Width: 1875 pixels
  ○ Height: 1832 pixels
  ○ 3 channels (RGB)

### 3.8 Folder: SWIR-HSI.zip

This .zip archive, named “SWIR-HSI.zip”, contains a folder named “SWIR-HSI”. The details of this folder are as follows:

• Folder containing the NIR-HSI data of the samples
  ○ Hyperspectral images are 3-dimensional images, with spatial dimensions x and y, and one spectral dimension, meaning that each pixel in the image constitutes a spectrum
• File names in format “sample_NNNN”
• Data of each sample is contained in two files conforming to the ENVI format^4^:
  ○ ․dat: the actual numerical hyperspectral data of the sample, in reflectance format
  ○ ․hdr: ASCII header file containing the metadata of the hyperspectral image
• Header information:
• samples = 379
  ○ lines = [variable: this is different for each sample, in the range 406 – 414]
  ○ bands = 288
  ○ header offset = 0
  ○ file type = ENVI Standard
  ○ data type = 4
  ○ interleave = bil
  ○ byte order = 0
  ○ wavelength = {953.04, […], 2547.64}

## 4 Experimental Design, Materials and Methods

In this section, we outline the used samples, data collection parameters, region of interest determination, mean spectrum and RGB value calculation, and outlier detection.

### 4.1 Sample collection

The samples for this work were collected by a partner company (Rester Oy) dealing with the end-of-life management of post-industrial garments. The partner company has information on the composition of each garment that enter their facility, obtained from the waste suppliers. The partner company collected these garments and the ground truth composition information of each garment. Each garment was labelled with an identification number. In addition, a log book containing metadata, including the colour and texture of the garments, as well as the ground truth composition information was collected.

We received the garments and the log book. In order to accommodate samples that could fit our measurement setup, we cut an approximately 10 cm by 10 cm piece from each garment, from the location deemed least worn out. This piece was the final sample that was used in our data collection.

### 4.2 Hyperspectral data collection and pre-processing

Details of the hyperspectral camera setup and data acquisition parameters are shown below. The hyperspectral images are contained in the folder “SWIR-HSI".

• Device: Specim SWIR 3
  ○ Optical characteristics:
    ■ Spectral range 1000-2500 nm
    ■ Spectral resolution (FWHM): 12 nm (mean)
    ■ Spectral sampling / pixel: 5.6 nm
    ■ F/# : F/2.0
  ○ Electrical characteristics:
    ■ Sensor: Cryogenically cooled MCT detector
    ■ Spatial pixels: 384
    ■ Spectral bands: 288
    ■ Frame rate: 24.1 fps
    ■ Integration time: 3.7 ms (Approx. 90% max signal)
    ■ Spatial binning: 1
    ■ Spectral binning: 1
    ■ Scanning speed: 7 mm/s
  ○ File type: ENVI
• Software: Lumo Scanner (proprietary software by Specim)
• Lighting: 6 quartz halogen lamps, directed at 45 degree angle symmetrically on the sample
• Background material of sample holder: black plastic
• Distance between camera and sample: 30 cm
• Raw signal data converted to reflectance using dark reference and white reference, gathered in tandem with sample measurements
• Dead pixels removed from the data
• Data in reflectance format

### 4.3 Photograph collection

Details of the device used for collecting red-green-blue (RGB) photographs of the samples are shown below. The photographs are contained in the folder “RGB”.

• Device: mobile phone Samsung Galaxy A52, SM-A525F/DS
• Camera settings
  ○ ISO 50
  ○ SPEED 1/20
  ○ FOCUS MANUAL, 0.6
  ○ WB 3100K
• Lighting: standard fluorescent lamps (Osram L58W/835)
• Background material of sample holder: black cotton
• Distance between camera and sample: 26 cm
• Data in RGB format

### 4.4 Region of interest and mean data

For each sample, we obtained both NIR-HSI and RGB data. In addition, in order to provide a single representative data point for each sample, we calculated mean spectrum and mean RGB values. This was done by viewing the images and selecting a rectangular region of interest (ROI) of each image. For the NIR-HSI images, the ROI was selected to be as large as possible but such that only the pixels that contain the sample are included. Thus, for certainty, we excluded the regions near the edges of the samples. The ROIs for the photographs were the same for all samples and were selected to include the largest area in the centre of all images; this was done in order to ensure the illumination is constant for all sample ROIs. The ROIs for hyperspectral data were unique, and are contained in the file “swir_mean_spectra_coordinates.csv”.

The ROIs act as annotations and thus can be used to extract pixels encompassing the sample from each image, both NIR-HSI and RGB. An example of this is included in the Python script. By selecting all the pixels in the ROI, the mean spectrum and mean RGB value of each sample was calculated. These are contained in the files “swir_mean_spectra.csv” and “rgb_mean_values.csv”, respectively.

### 4.5 Example of data

**Fig. 2:**
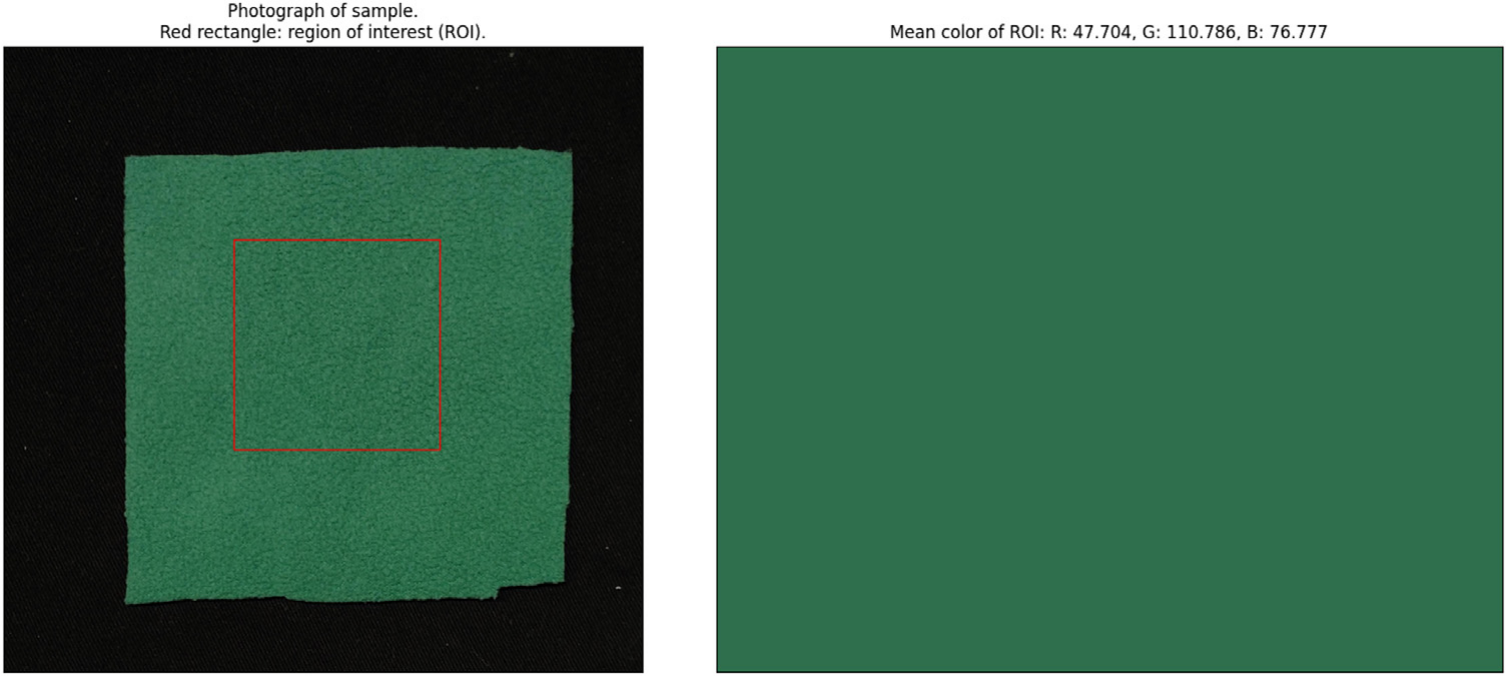
Illustration of the RGB data of the example sample: RGB image and visualization of ROI (left), and the mean RGB value of the ROI (right).

**Fig. 3:**
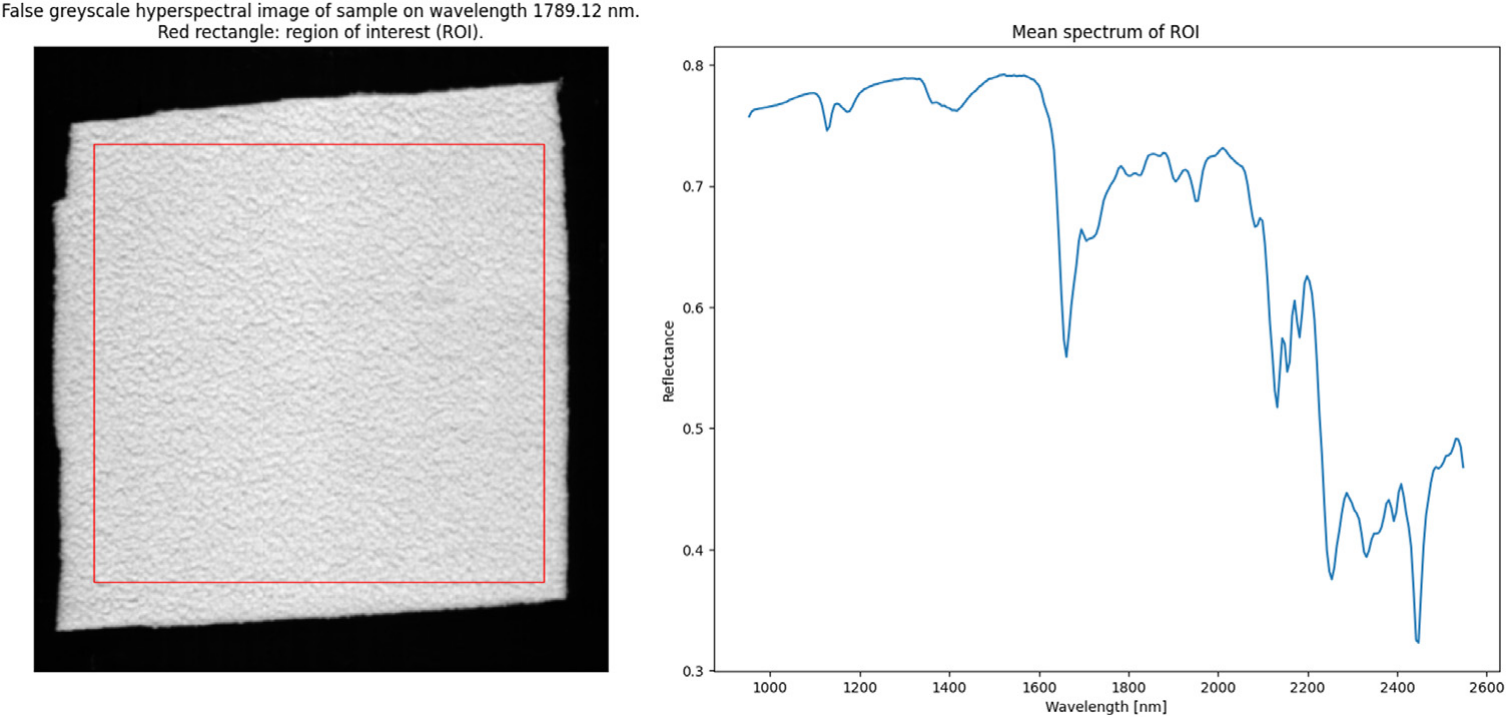
Illustration of the NIR-HSI data of the example sample: false grayscale NIR-HSI image and visualization of ROI (left), and the mean NIR spectrum of the ROI (right).

Here, we provide example data for a single sample in the dataset. We selected the sample labelled “sample_15-1” for this. The ground truth of the sample is shown in Table 1. A visualization of the RGB image, the ROI, and the mean RGB value extracted from the ROI, is shown in Fig. 2. Finally, a visualization of the NIR-HSI image, the ROI, and the mean NIR spectrum extracted from the ROI, is shown in Fig. 3.

**Table 1:** Ground truth of the example sample in tabular format.

	sample_15-1
Unknown?	
Polyamide [%]	
Viscose [%]	
Lyocell [%]	
Modacrylic [%]	
Polyacrylic [%]	
Wool [%]	
Carbon fibre [%]	
Polyurethane [%]	
Elastane [%]	
Polyester [%]	100.0
Cotton [%]	
Colour	Green
Texture	Fleece
Extra information

### 4.6 Outlier detection for hyperspectral data

For outlier detection, we utilized the same spectral pre-processing and regression modelling chain as outlined in section *Example use case: Prediction of natural fibre content in the samples*. The process was as follows: For each sample, we obtained the predicted natural fibre composition from the regression model. We calculated the absolute difference of the predicted and the ground truth value for all samples. Further, we calculated the standard deviation of these differences in the dataset. The samples whose difference was greater than 3 standard deviations were deemed as an outlier. The outliers were temporarily removed from the dataset.

The process above was repeated three times, with each time removing samples from the temporary dataset. At the end, 11 samples in total were deemed as outliers. These are marked with the label “outlier” in the ground truth metadata file (ground_truth_final.csv), into the column “Unknown or outlier?”.

## 5 Using the Dataset

In this section, we outline possible use cases for the dataset. We also include two example analytics workflows using machine learning for classification and regression, including the results obtained therein.

### 5.1 Envisioned use cases

Since hyperspectral datasets for optical identification are rare, we expect multiple different use cases for this dataset. These are enumerated below. Two of these cases are included as an example in this paper (see sections *Example use case: Prediction of natural fibre content in the samples* and *Example use case: Classification of textiles based on colour*).

• Classification of textiles based on material composition
• Classification of textiles based on colour (included as an example in this paper)
• Prediction of natural fibre content in the samples (included as an example in this paper)
• Image segmentation based on spectral information
• Image segmentation based on colour information
• Developing and optimizing machine learning algorithms for spectral classification
• Developing and optimizing frameworks for spectral waveband selection for machine learning algorithms
• Use as NIR reference spectral library for different materials, e.g. using the mean spectra of samples
• Huge spectral library of different materials: pixels in ROI in the hyperspectral images
• Demonstration material for e.g. university courses on hyperspectral imaging, data analysis, and signal processing

### 5.2 Example use case: prediction of natural fibre content in the samples

Natural fibres in the dataset constitute of cotton, wool and lyocell^5^. Thus, the percentage of these components in each sample was summed to form the ground truth for modelling. The samples labelled as unknown or outlier were removed from the dataset.

In this use case, we utilized the mean NIR spectra of the samples (file “swir_mean_spectra.csv”). Spectral pre-processing was done as follows:

• Data conversion from reflectance to absorbance
• Savitzky-Golay filtering (2^nd^ degree polynomial, window size 9)
• Cutting the spectral range to include wavelengths 1411 – 2536 nm
• Standard normal variate for data normalization

For regression, we utilized partial least squares. The target value for regression is the natural fibre composition, which was predicted using the spectral data. We iterated the number of latent variables in the partial least squares model in the range 1 – 10. We utilized a leave-one-out cross-validation scheme, such that for each latent variable in the aforementioned range, we train a model with all but one sample and predict the composition of the left-out sample; this is repeated such that each sample is left out once and thus a predicted value is obtained for each sample.

**Fig. 4:**
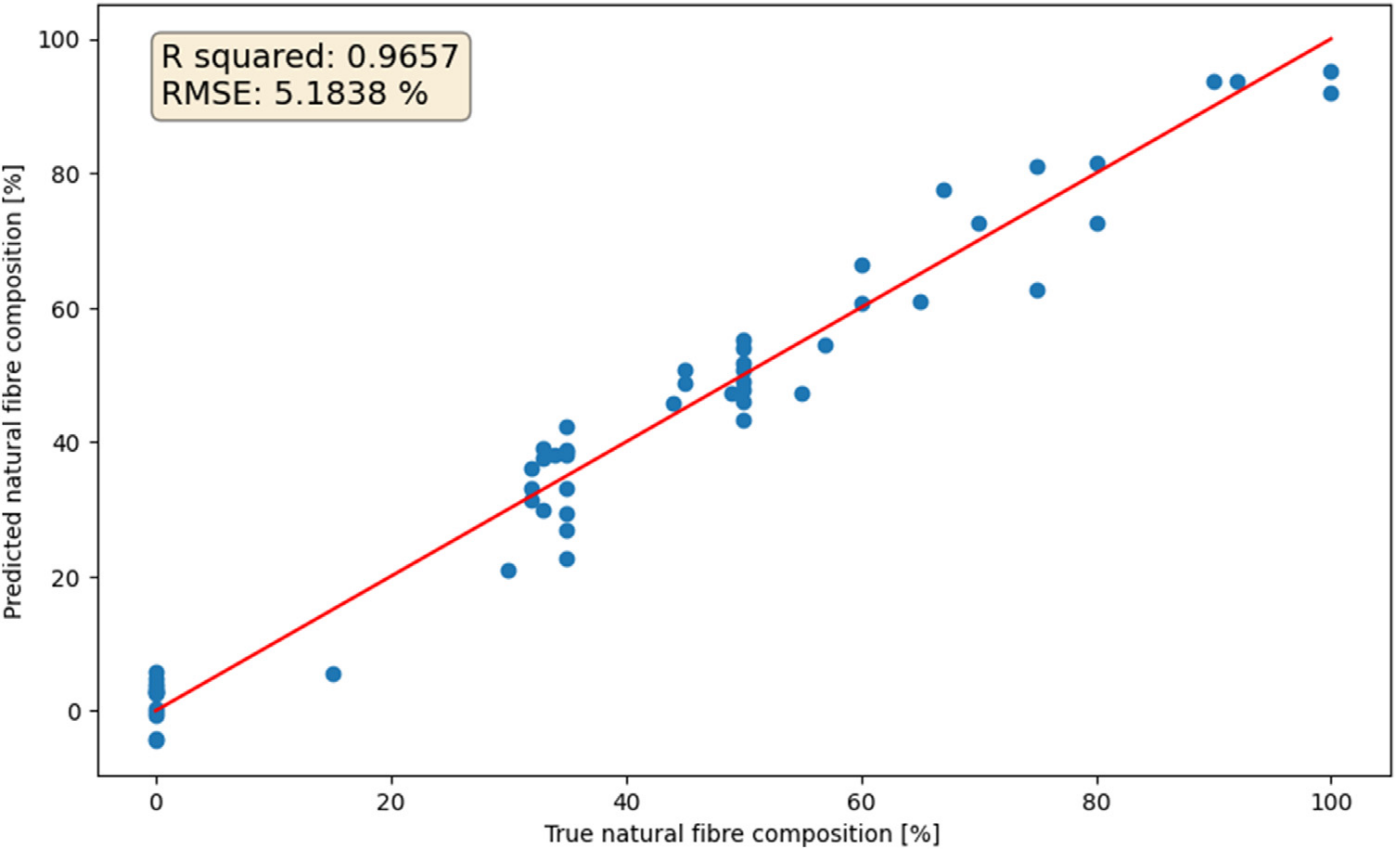
Cross-validation results of our natural fibre content prediction model.

We obtained the best results using 4 latent variables; the root mean squared error was 5.18% and coefficient of determination 0.9657, which indicate excellent results. A scatter plot of the prediction is shown in Fig. 4. As can be seen, most of the data points align well with the best fit line. However, there was large variation between samples. The maximum difference between the predicted and the actual value was 12.4%, while the minimum difference was 0.16%. These can arise from the fact that the blend percentage can change during the lifetime of the garment [6], as well as possible errors in sample labelling, although care was taken to remove outliers, as explained in the section *Outlier detection for hyperspectral data*.

In order to provide robustness, we also performed the same framework outlined above but with using K-fold cross-validation instead of leave-one-out cross-validation. We utilized different values for K, namely 3, 5, and 7. The results of these approaches are found in *Supplementary Material A*. In short, the results using K-fold cross-validation do not significantly differ from those obtained with leave-one-out cross-validation, providing validity to our approach.

### 5.3 Example use case: classification of textiles based on colour

We utilized the ground truth information of the colour of each sample to build a classification model that identifies black textiles based on RGB data. For this, we utilized the mean RGB value of each sample (file “rgb_mean_values.csv”). We converted these RGB values to CIELAB colour space values. We utilized Decision Tree classifier as the classification model. To gauge the performance of the model, we used leave-one-out cross-validation scheme for the data. Note that here, as opposed to the analysis in the previous section (*Example use case: Prediction of natural fibre content in the samples*), no samples were left out due to being an outlier, as the ground truth colour information could be verified visually.

**Fig. 5:**
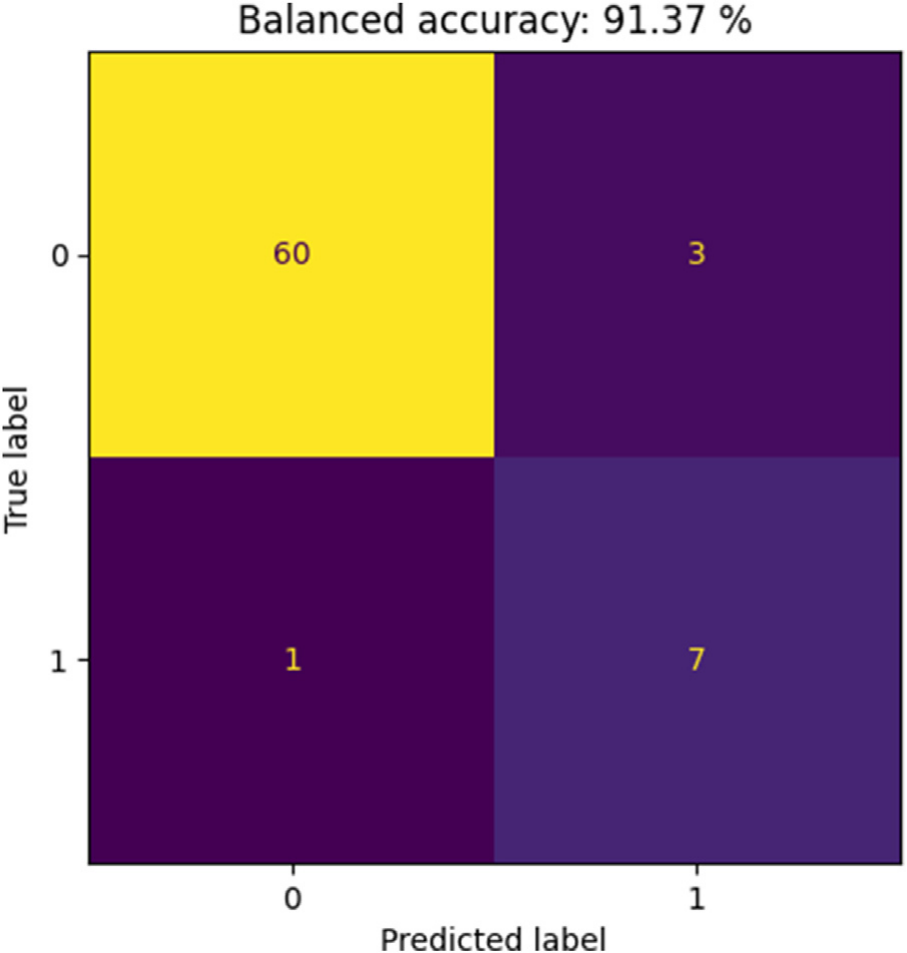
Cross-validation results of our colour classification model: classification confusion matrix.

The classification results are shown in Fig. 5. We obtained 91.37% balanced accuracy, with only 4 samples misclassified, indicating very good results. Most of the misclassified samples were false positives (3), with only 1 false negative, i.e. the classification model is slightly skewed to incorrectly predict the sample as black. The false negative sample was sample “sample_34-1”, which according to the ground truth information is black but “very glossy”. This most likely explains the erroneous classification. The three false positives were samples “sample_9-1”, “sample_25-1”, and “sample_26-1”, whose ground truth information reveals that they are dark coloured. As such, a more precisely calibrated algorithm might detect the difference between these dark coloured and black samples.

As previously for the natural fibre content prediction, we also performed K-fold cross-validation in order to test the robustness of our approach. Here, we utilized the same values for K, namely 3, 5, and 7. The results of these approaches are found in *Supplementary Material B*. In short, the results using K-fold cross-validation are identical to the ones obtained using leave-one-out cross-validation, providing validity to our approach.

## Limitations

The main limitation of this dataset is that the ground truth fibre composition of each garment was not verified using chemical means. The samples were acquired and labelled by our partner company according to the information provided by the manufacturer. It is possible that human errors could have been made along the way, or that the samples contain unreported coatings that affect the spectral response. Indeed, our outlier detection revealed that the provided composition information of 11 samples may be erroneous. Thus, these outlier samples should be used with care. Moreover, as the ground truth composition information is acquired from the manufacturer based on the manufacturing phase, the exact fibre composition at current time may be different. This is due to the fact that blend percentage of samples may change due to different wear rates of the fibre components, leading to changes in spectra as well [6]. However, as seen in our regression model results (section *Example use case: Prediction of natural fibre content in the samples*), we obtained very good regression results, indicating that the change is not very large. Finally, the number of different pure fibre types in the dataset is rather limited, pointing to requirements for future work.

## Ethics Statement

The authors have read the ethical requirements of the journal and confirm that the current work does not involve human subjects, animal experiments, or any data collected from social media platforms.

## CRediT Author Statement

*Please outline the contributions of each co-author, using the categories listed on this webpage.*

**Tuomas Sormunen:** Writing – original draft, Investigation, Software, Data curation, Methodology, Validation, Formal analysis; **Ella Mahlamäki:** Investigation, Writing – review & editing, Conceptualization, Data curation; **Satu-Marja Mäkelä:** Conceptualization, Funding acquisition, Writing – review & editing; **Mikko Mäkelä:** Writing – review & editing, Funding acquisition, Methodology, Conceptualization, Supervision.

## Data Availability

ZenodoOpenTextile-NIR: Near-infrared hyperspectral imaging and photography dataset for optical identification of textiles (Original data).

## Appendix Supplementary materials

Image, application 1

## Footnotes

1 Version available at: https://www.python.org/downloads/release/python-31011/ (accessed 20.1.2026).

2 Version available at https://www.piwheels.org/project/pip/ (accessed 20.1.2026)

4 More info on ENVI file format here: https://www.nv5geospatialsoftware.com/docs/ENVIImageFiles.html (accessed 20.1.2026)

## Footnotes

3 .zip archives can be extracted e.g. with WinZip: https://www.winzip.com/en/ (accessed 20.1.2026)

5 Strictly speaking, lyocell is a semi-synthetic fibre, but is made from natural sources (regenerated cellulose). Thus, it’s spectral profile is similar to cotton.
