# Supplementary material for: Introducing OpenTextile-NIR: Near-infrared hyperspectral imaging and photography dataset for optical identification of textiles
Source: Data Brief. 2026 Feb 9;65:112559. doi: 10.1016/j.dib.2026.112559 (PMC12925456; doi:10.1016/j.dib.2026.112559)
Supplement: Supplementary file 1 [file mmc1.docx]

**Supplementary material A: K-fold cross-validation of our natural fibre content regression model**

In the manuscript, in Section *Example use case: Prediction of natural fibre content in the samples*, we outlined our method for building the machine learning model for natural fibre content prediction. To validate the approach, we utilized leave-one-out cross-validation scheme. In order to provide more information on the validity of our approach, here we perform K-fold cross-validation for the model using three different values of K, namely 3, 5, and 7. Otherwise, we utilize the same framework for data pre-processing, latent variable iteration and regression. The results for each value of K are shown in the respectively named subsections below. As can be seen from these sections, the results obtained with each values of K are very similar to the ones using the leave-one-out cross-validation scheme, supporting the validity of our approach.

K-fold cross-validation of natural fibre content, K = 3

For K = 3, we obtained the best results using 4 latent variables. The root mean squared error value was 5.16%, and the coefficient of determination was 0.966, indicating excellent results. A scatter plot of the prediction is shown in Supplementary Figure 1. These results are very similar but slightly better to the ones obtained using the leave-one-out cross-validation scheme.


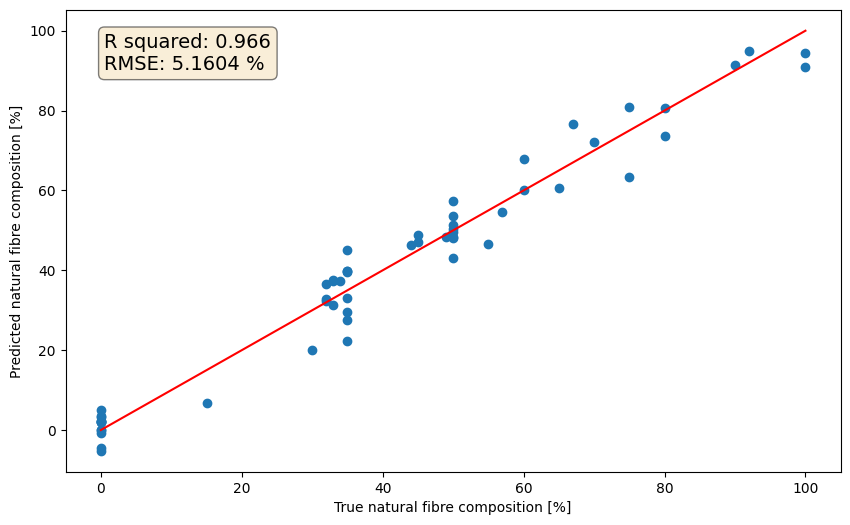


Supplementary Figure 1. K-fold cross-validation results of our natural fibre content prediction model (K = 3).

K-fold cross-validation of natural fibre content, K = 5

For K = 5, we obtained the best results using 4 latent variables. The root mean squared error value was 5.06%, and the coefficient of determination was 0.967, indicating excellent results. A scatter plot of the prediction is shown in Supplementary Figure 2. These results are very similar but slightly better to the ones obtained using the leave-one-out cross-validation scheme.


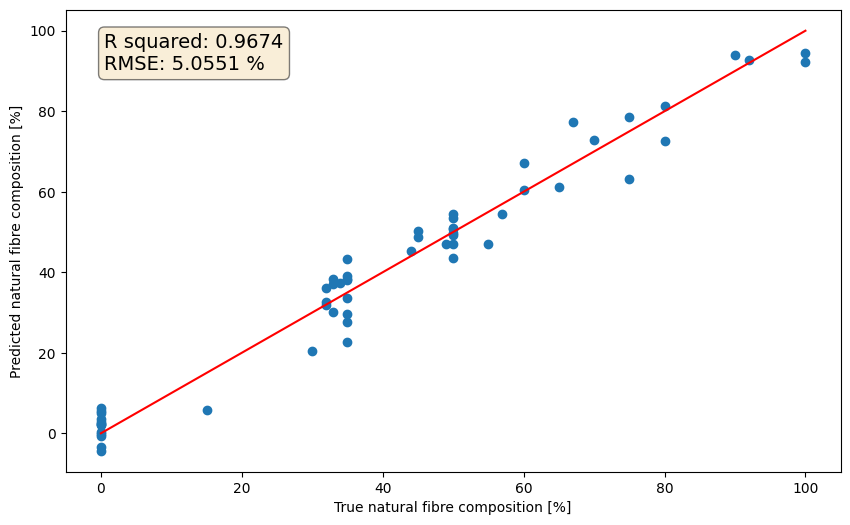


Supplementary Figure 2. K-fold cross-validation results of our natural fibre content prediction model (K = 5).

K-fold cross-validation of natural fibre content, K = 7

For K = 7, we obtained the best results using 3 latent variables. The root mean squared error value was 5.27%, and the coefficient of determination was 0.9646, indicating excellent results. A scatter plot of the prediction is shown in Supplementary Figure 3. These results are very similar but slightly poorer to the ones obtained using the leave-one-out cross-validation scheme. In addition, the iteration yielded a lower number of latent variables for which the lowest root mean squared error was obtained.


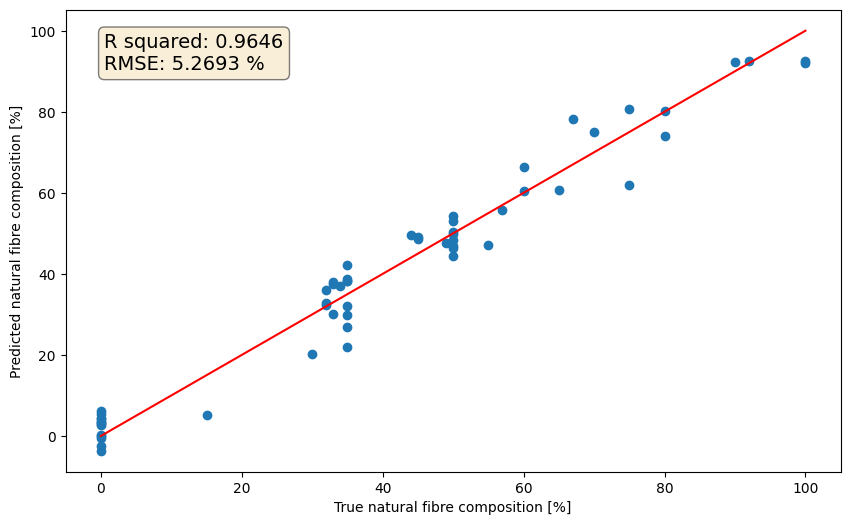


Supplementary Figure 3. K-fold cross-validation results of our natural fibre content prediction model (K = 7).

**Supplementary material B: K-fold cross-validation of our colour classification model**

In the manuscript, in Section *Example use case: Classification of textiles based on colour*, we outlined our method for building the machine learning model for colour-based classification. To validate the approach, we utilized leave-one-out cross-validation scheme. In order to provide more information on the validity of our approach, here we perform K-fold cross-validation for the model using three different values of K, namely 3, 5, and 7. Otherwise, we utilize the same framework for data pre-processing and classification.

The results for all K-fold cross-validation schemes (3, 5, and 7) were exactly the same. The obtained balanced accuracy was 91.37%, and the confusion matrix is seen in Supplementary Figure 4. As can be seen, the results are identical to the ones obtained with our leave-one-out cross-validation scheme, supporting the validity of our approach.


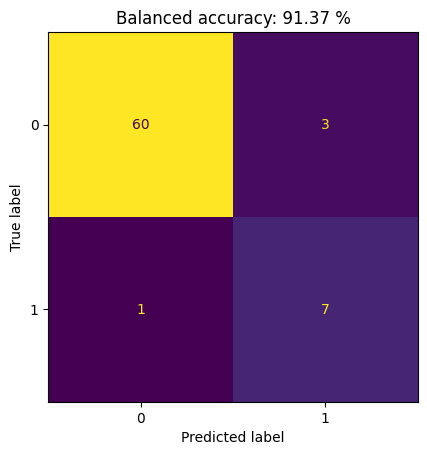


Supplementary Figure 4. K-fold cross-validation results of our colour classification model. The same confusion matrix was obtained for all values of K (3, 5, and 7).
